# Data on the effect of *Pseudomonas stutzeri* E25 and *Stenotrophomonas maltophilia* CR71 culture supernatants on the mycelial growth of *Botrytis cinerea*

**DOI:** 10.1016/j.dib.2018.01.023

**Published:** 2018-01-28

**Authors:** Daniel Rojas-Solís, Gustavo Santoyo

**Affiliations:** Instituto de Investigaciones Químico-Biológicas, Universidad Michoacana de San Nicolás de Hidalgo, Morelia 58030, Michoacán, Mexico

**Keywords:** Plant growth-promoting bacterial endophytes, Volatile organic compounds, Culture supernatants

## Abstract

Plant growth-promoting bacterial endophytes (PGPBEs) produce volatile and diffusible compounds that inhibit phytopathogens (Santoyo et al., 2016) [1]. A recent work by Rojas-Solis and colleagues [2] demonstrated the antifungal effect of volatile organic compounds exerted by the *Pseudomonas stutzeri* E25 and *Stenotrophomonas maltophilia* CR71 endophytes, highlighting the production of sulfur-containing compounds such as the antimicrobial volatile dimethyl disulfide (DMDS). The data presented in this article include the effect of two culture supernatants from the same strains, E25 and CR71, on the mycelial growth of the gray mold phytopathogen *Botrytis cinerea*. These data may help to further evaluate the specific compounds produced by endophyte isolates E25 and CR71 with antifungal activity. This article is submitted as a companion paper to Rojas-Solís et al. (2018) [2].

**Specifications Table**

**Table:** 

Subject area	Biology
More specific subject area	Biological Control
Type of data	Graph, figure
How data was acquired	Plate bioassays
Data format	Raw data statistically analyzed
Experimental factors	The data concern filtered overnight bacterial cultures.
Experimental features	The experimental design included potential antifungal activity of bacterial culture supernatants.
Data source location	Morelia, México
Data accessibility	Data is within this article

**Value of the data**

• The data show the effect of culture supernatants of *Pseudomonas stutzeri* E25 and *Stenotrophomonas maltophilia* CR71 on the mycelial growth of the gray mold phytopathogen *Botrytis cinerea.*
• The data highlight the weak antifungal effect of diffusible compounds produced by bacterial endophytes *Pseudomonas stutzeri* E25 and *Stenotrophomonas maltophilia* CR71.
• The data are useful to further explore the compounds responsible for antifungal action.

## 1 Data

PGPBEs can produce either antifungal diffusible or volatile compounds [1, 2].

These data show the direct effect of *P. stutzeri* E25 and *S. maltophilia* CR71 culture supernatants on the mycelial growth of the phytopathogen *B. cinerea* using culture assays on potato dextrose agar (PDA) plates (Table 1). Values are presented as the means±standard errors of three replicates from repeated experiments. Different letters in each column indicate significant (*p*<0.05) differences according to the least significant difference test.

**Table 1:** Antifungal activities of *Pseudomonas stutzeri* E25 and *Stenotrophomonas maltophilia* CR71 culture supernatants on mycelial growth of *Botrytis cinerea*.

Treatment	Mycelial growth area (cm^2^)	Mycelial growth inhibition (mm)
Control	45.96±8.86 ab	Not detected
E25 (0.1X)	41.69±11.05 ab	7.28±0.47 a
E25 (0.5X)	44.47±5.10 ab	7.83±0.52 a
E25 (1X)	32.47±3.08 a	7.3±0.25 a
CR71 (0.1X)	50.42±8.86 b	7.0±0.59 a
CR71 (0.5X)	30.07±3.48 ab	6.75±0.39 a
CR71 (1X)	47.79±7.95 ab	6.95±0.44 a

## 2 Experimental design, materials and methods

The culture supernatants of isolates *Pseudomonas stutzeri* E25 and *Stenotrophomonas maltophilia* CR71 were tested for antifungal activities against *B. cinerea* through plate bioassays. Overnight cultures were grown (O.D. 1.2) on liquid nutrient media and filter-sterilized using 0.2-µm membranes. Then, 1 mL of media at 1×, 0.5× and 0.01× concentrations were inoculated via flooding onto PDA plates and allowed to dry in a laminar flow cabinet. A 4-mm mycelial plug (from a freshly pre-grown culture of *B. cinerea*) was deposited in the center of each plate and incubated in darkness at 30 °C. Mycelial growth inhibition (mm) and mycelial growth area (cm^2^) were measured at day 6. The mycelial growth area (cm^2^) was determined using ImageJ software.
